# Neuropsychiatric Lupus and Lupus Nephritis Successfully Treated with Combined IVIG and Rituximab: An Alternative to Standard of Care

**DOI:** 10.1155/2022/5899188

**Published:** 2022-08-28

**Authors:** Mohamed M. Cheikh, Abdullah K. Bahakim, Moayad K. Aljabri, Saad M. Alharthi, Sanad M. Alharthi, Abdullah K. Alsaeedi, Saad F. Alqahtani

**Affiliations:** ^1^Department of Medicine, Fakeeh College for Medicine Science, Jeddah, Saudi Arabia; ^2^Internal Medicine Department, Doctor Soliman Fakeeh Hospital, Jeddah, Saudi Arabia; ^3^Department of Medicine and Surgery, College of Medicine, Umm Al-Qura University, Makkah, Saudi Arabia

## Abstract

Systemic lupus erythematosus (SLE) is a chronic autoimmune disease with unpredictable course and flares. The clinical manifestation can vary from mild to severe and life-threatening disease. Infection is the primary cause of mortality in hospitalized SLE patients. There is a paucity of evidence to support the co-management of SLE with major organ involvement and sepsis. We describe the clinical response of a 35-year-old male diagnosed with SLE; then, he developed severe sepsis and a flare of SLE with major organ involvement including lupus nephritis (LN), myocarditis, and neuropsychiatric systemic lupus erythematosus (NPSLE). Based on the patient's condition, a treatment dilemma was encountered, and after a multidisciplinary meeting, the decision was made to use a combination of rituximab (RTX), intravenous immunoglobulin (IVIG), and pulse steroid. Shortly, the patient's condition started to improve, and his symptoms were resolved. In conclusion, our clinical case suggests that combined RTX, IVIG, and pulse steroid seem to be effective and safe in achieving clinical response, thus representing a good choice for managing severe SLE flares in sepsis.

## 1. Introduction

Systemic lupus erythematosus (SLE) is a relapsing-remitting systemic autoimmune disease with a strong female predominance and complex etiologies [1]. The clinical presentation can range from mild or “organ limited” to major organ involvement and life-threatening disease. Mild manifestations can include arthritis (85%), skin rashes (71%), Raynaud's (37%), nonscarring alopecia (31.5%), fever (31%), oral/nasal ulcers (26%) whereas major organ involvement includes renal disease (41.5%), neuropsychiatric (17%), hematological (12%), pulmonary (7%), and cardiac (3%) [2, 3]. Neuropsychiatric SLE (NPSLE) refers to a series of neurological and psychiatric symptoms directly related to SLE that involve the central and peripheral nervous system (aseptic meningitis, CVA, acute confusional state, mood and anxiety disorders, psychosis, Guillain–Barre syndrome, and myasthenia gravis) [4]. Lupus nephritis (LN) is a type of glomerulonephritis mediated by immune complex deposition at glomerular sites and represents one of the severe major organ involvements in SLE. LN is histologically categorized into six distinct patterns with various manifestations and severities [5]. Cardiac involvement is one of the significant causes of SLE mortality, including pericarditis, myocarditis, Libman–Sacks endocarditis, and coronary vasculitis. Myocarditis is the most specific feature of myocardial involvement, and its symptoms can start with fever, chest pain, and dyspnea and may progress into heart failure and dilated cardiomyopathy [6, 7]. High-dose glucocorticoid (GC) alone or plus immunosuppressive therapy (IS) such as intravenous cyclophosphamide (CYC) is the standard therapy for NPSL, LN, and lupus myocarditis [8, 9]. Infection is the leading cause of death in hospitalized SLE patients; the risk of infection is associated with high disease activity, renal involvement, leucopenia, thrombocytopenia, and treatment with high-dose GC and IS agents [8, 10]. Treating a patient with a flare of autoimmune diseases like SLE and sepsis might be challenging. Here we present a case of SLE with involvement of three major organs in the context of infective endocarditis and sepsis that were successfully treated with pulse steroid, rituximab (RTX), and intravenous immunoglobulin (IVIG).

## 2. Case Presentation

A 35-year-old black race male with a past medical history of hypertension and cardiomyopathy presented on 29 June 2021 to the outpatient department (OPD) with fatigability, weight loss of more than 10 kg over 1 year, subjective fever, night sweat, anorexia, malaise, oral ulcer, body aches, and no evidence of arthritis. On examination, mildly elevated blood pressure, marked proximal and distal muscle weakness, and enlargement of the subclavian and cervical lymph nodes were noted. Initial laboratory test result showed normocytic normochromic anemia, positive antinuclear antibody (ANA) 262.2 (by ELISA technique), anti-SM antibody 693.50 CU, anti-ribonuclear protein 643.8 CU, C3 0.13 g/l, and C4 0.01 g/l. Computed tomography (CT) scan of the chest, abdomen, and pelvis with intravenous (IV) contrast was obtained and showed enlarged lymph node groups above and below the diaphragm associated with hepatosplenomegaly. The patient was admitted, and an axillary excisional lymph node biopsy was done. The initial report was suggestive of follicular and paracortical hyperplasia. Due to the severity of his severe constitutional symptoms and multiple lymph node enlargements, which might be associated with lymphoma, the biopsy was sent for a second opinion which came back with negative findings of lymphoma and confirmed a benign finding that includes follicular and paracortical hyperplasia. The diagnosis of SLE was established based on the Systemic Lupus International Collaborating Clinics (SLICC) criteria (oral ulcer, positive ANA, anti-Ds-DNA, anti-SM antibody, and low *C*3). The patient was started on prednisolone 30 milligrams (mg) daily and hydroxychloroquine (HCQ) 400 mg daily and discharged home.

After six days, the patient presented to the emergency room with severe musculoskeletal (MSK) manifestations (arthritis of both wrists, elbow, metacarpophalangeal (MCP) joints, proximal interphalangeal (PIP) joints, and knee joint). He was admitted to the floor based on his MSK symptoms and started on intravenous 1 mg/kg of methylprednisolone and painkillers. The patient also had cardiomyopathy manifestations in the form of exertional dyspnea and lower limb edema, which was confirmed with an echocardiogram (ECHO) that showed mild systolic dysfunction (ejection fraction of 45–50%). The ECHO findings upon admission were similar to the previous baseline ECHO that was done six months prior to this presentation.

During admission, the patient started to show signs of ongoing sepsis in the form of frequent spikes of high-grade fever and an increase in leukocytes (17.53 × 103/*μ*l) and C-reactive protein (232.70 mg/L). He was started on broad-spectrum antibiotics, and septic screen that includes cultures of blood, urine, and sputum, chest X-ray, and knee joint aspiration was done. The blood culture showed persistent Gram-negative *Salmonella* non-Typhi and MSSA bacteremia for three days, and the source of MSSA bacteremia was most likely septic arthritis due to the growth of MSSA in the synovial fluid cultur[[parms resize(1),pos(50,50),size(200,200),bgcol(156)]] necessitating admission to the intensive care unit (ICU) for vasopressors and mechanical ventilation. Continuous renal replacement therapy (CRRT) was introduced after the deterioration in the patient renal condition and he became anuric; the urine albumin to creatinine ratio was 1560 mg/g. Although his AKI was multifactorial, including contrast nephropathy, sepsis, and possible glomerulonephritis, a kidney biopsy was taken to rule out lupus nephritis. The biopsy showed diffuse global lupus nephritis with active lesion class IV.

Transthoracic echocardiogram (TTE) and transesophageal echocardiogram (TEE) did not show definitive valve vegetation, and CT pulmonary angiography was done and it ruled out pulmonary embolism; however, it showed right lower lobe nodular consolidation. Three days later, CT abdomen and pelvis with contrast was done, and it showed no evidence of bowel perforation and slight regression of the right lower lobe nodular consolidations with new central cavitation was noted; findings suggest infectious process/septic emboli. The patient was seen by infectious disease (ID) team, and according to Duke criteria for infective endocarditis (IE), the patient met one major criteria (typical organism from 2 separate blood cultures, persistently positive blood cultures drawn >12 hours apart) and two minor criteria (findings of infectious process/septic emboli and fever). An ophthalmology examination showed no Roth spot. The patient was started on meropenem 1 g IV three times a day (TID) for eight weeks, covering *Salmonella* and MSSA.

**Figure 1:**
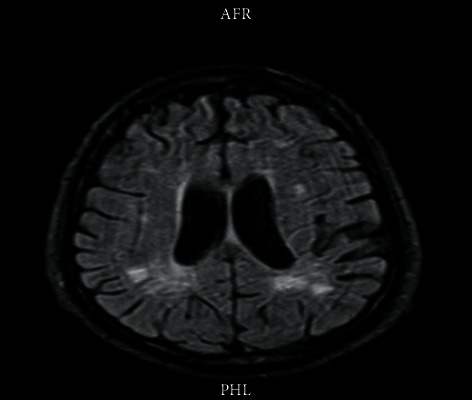
Brian MRI showing bilateral cerebral subcortical, deep white matter, and centrum semioval bright signal foci in *T*2/weighted images.

**Figure 2:**
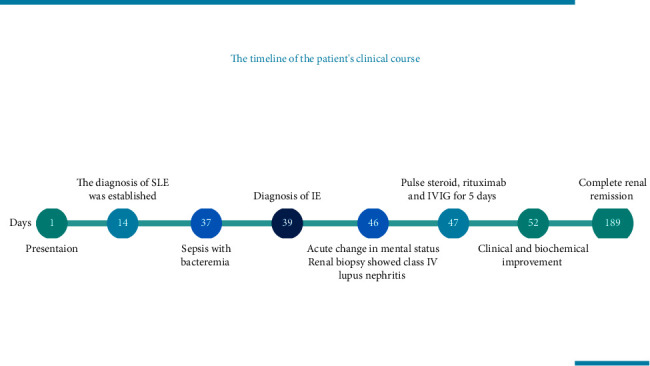
The timeline of the patient's clinical course. Days are represented in circles and clinical events are arranged chronologically.

A week later, the patient started to recover from his septic shock, he was extubated, and his blood pressure was stabilized without vasopressors. However, he developed new symptoms; he was found to have an acute change in his mental status in the form of visual hallucination, confusion, elated mood, and disorientation to the time and place. Magnetic resonance imaging (MRI) of the brain with contrast showed bilateral deep white matter enhancement (Figures 1 and 2). Lumbar puncture (LP) was done to exclude infectious causes with film array meningitis panel and culture, which were negative. However, he had albuminocytological dissociation, high cerebrospinal fluid (CSF) protein (113.5 mg/dl), and IgG without pleocytosis, which indicated an intracranial inflammatory process. The patient was diagnosed with neuropsychiatric manifestations of lupus; this was supported by very active serology with an anti-dsDNA antibody of more than 669 IU/ml and very low C3 and C4 complement despite high doses of methylprednisolone 1 mg/kg on the preceding seven days. The patient also had a drop in his ejection fraction from 40% to 30%, with high troponin suggesting myocarditis.

Cyclophosphamide was not given because the patient was recovering from severe sepsis that required ICU admission, vasopressors, and intubations. However, weighing risks and benefits and after a multidisciplinary meeting with ICU, infectious disease, rheumatology, and nephrology teams, the decision was made to give him a combined IVIG of 0.4 g/kg/day for five days and RTX 500 mg once with pulse steroid therapy 1 g/day for five days. This regimen was started after seven days of the first negative blood culture.

After five days, the patient's condition started to improve. The patient's hallucinations subsided, and he was oriented and less confused with intact short-term and long-term memory. The clinical improvement was accompanied by improvement of biochemical markers and other indicators of the severity of the patient's condition. Anti-dsDNA upon admission was >666.90 IU/ml and then declined to 55.3 IU/ml, and C3 and C4 upon admission were (0.13, 0.01) g/l and then increased to (0.79, 0.16) g/l, respectively (Table 1).

Three weeks later, the patient was started on mycophenolic acid (MMF) 500 mg gradually to be increased to 1 g twice a day (BID) besides 1 mg/kg prednisolone for four weeks and then tapered down with a reduction of 10 mg per week and hydroxychloroquine (HCQ) 400 mg in order to maintain this remission and control other system manifestations.

After four months, there was a marked improvement in his MSK manifestations and neuropsychiatric symptoms (cognitive and conscious levels improved). Regarding lupus nephritis, he achieved complete remission (albumin to creatinine ratio decreased from 1560 to 150). In order to achieve full disease control, he was given belimumab 200 mg subcutaneously every week. In addition, his decompensated heart failure symptoms improved, and his repeated TTE showed that his ejection fraction is back to the baseline of 40%. He is taking anti-failure therapy, including angiotensin-converting enzyme inhibitors (ACE-I) and beta-blockers, and continuing HCQ 400 mg, double-strength tablet trimethoprim 160, sulfamethoxazole 800 mg for pneumocystis pneumonia prophylaxis, and prednisone 10 mg/week.

## 3. Discussion

In this case report, a challenging clinical scenario was encountered. First, our patient was diagnosed with SLE; then, he developed severe sepsis and a flare of SLE with major organ involvement (LN, NPSLE, and myocarditis).

The substantial immune dysfunction among patients with SLE makes them more susceptible to infection. Both disease activity and therapy play a crucial role in infection development. Infections can be confusing to distinguish from disease flare-ups in SLE patients. The death rate in SLE patients with infection is five times higher than the general population. Growing literature evidence highlights the pivotal role of infections in the exacerbation of SLE [11].

The treatment option depends on existing disease manifestations, patient age, safety concerns, and cost. Currently, hydroxychloroquine is considered mandatory in managing SLE and is recommended throughout the entire course of the disease. Furthermore, glucocorticoids (GCs) provide the most immediate anti-inflammatory impact of all immunosuppressive treatments and are considered the more widely utilized therapy to manage mild-severe flares of SLE [12]. Even more critical, cyclophosphamide (CYC) is used in organ-threatening diseases (specifically renal, neuropsychiatric, or cardiopulmonary) and only as a rescue treatment in refractory non-major organ involvements. Also, it is imperative to highlight the risk of CYC therapy use during infections [8].

Our patient came with sepsis and a flare of SLE with involvement of three major organs, which was a puzzling scenario due to the lack of formal guidelines for co-management of sepsis and involvement of three major organs. In our opinion, since there is no evidence to support one over the other regimen in such a case, a combination of RTX, IVIG, and pulse steroid administrated was well tolerated in our patient and may be a better decision due to its better side effect profile; nevertheless, this management plan must be taken based on experts' opinion.

Nowadays, RTX is solely used off-label in SLE patients with severe renal or extrarenal disease refractory to other IS agents or contraindications to these drugs [8]. While giving IVIGs in SLE patients has inadequate evidence for their efficacy and is derived from case reports and case series, existing indications suggested IVIGs in SLE for severe cases not responding to conventional immunosuppressive agents, patients with active SLE, and concomitant infection [1].

Throughout the literature review, we found various cases reported RTX and IVIGs. This combination showed efficacy and safe therapeutic response in managing complicated SLE. To begin with, a 41-year-old Caucasian male with a history of SLE had myocarditis; then, he developed refractory cardiogenic shock treated with RTX, IVIG, and steroids, which resulted in slight improvement of the left ventricle function [13].A Previous case report described a patient with SLE and recurrent diffuse alveolar hemorrhage (DAH) who did not respond to standard treatment and consequently received RTX and IVIG successfully [14]. Moreover, Costa et al. demonstrated a clinical case of a 32-year-old woman with lupus and fever of unknown origin, who developed unusual complications, including visceral leishmaniasis, macrophage activation syndrome (MAS), and DAH that was started on RTX, IVIG, and steroid and reported a good response [15].

In addition to previous cases, a case of a 35-year-old woman with SLE who developed hypoprothrombinemia-lupus anticoagulant syndrome achieved a sustained remission following treatment with RTX, IVIG, and prednisolone [16]. Lastly, a 64-year-old man presented with relapsed NPSLE, and a combination of IVIG and RTX was started. Consequently, his neuropsychiatric symptoms subsided, and hisserological testing revealed decreased disease activity [17].

In conclusion, our clinical case suggests that the RTX, IVIG, and pulse steroid combination therapeutic scheme appears to be safe and successful in achieving a clinically significant response, thus representing a good choice for treating severe SLE manifestations in sepsis including LN, NPSLE, and myocarditis. Of course, it would be hard to attribute the response to one agent more than the others, but this strategy effectively reduced inflammation and prevented imminent death. Furthermore, to our knowledge, this is the first case with this dilemma in treatment that was successfully treated.

## Figures and Tables

**Table 1:** Laboratory data through the course.

Variable	On presentation to the OPD	During sepsis	After combined rituximab and IVIG	Reference range
WBC count (10^3^/*μ*l)	3.84	17.53	4.90	4.5–11
Deferential count				
Neutrophils (%)	51.30	91.50	46.50	35–80
Lymphocytes (%)	42.70	5.00	39.00	24–44
Monocytes (%)	4.20	3.20	12.90	4.7–12.5
Eosinophils (%)	1.80	0.10	1.40	0–4
Basophils (%)	0.00	0.20	0.20	0–1
RBC count (10^6^/*μ*l)	3.28	3.67	5.73	4.3–5.7
Hemoglobin (g/dl)	8.50	10.10	14.60	13.2–17.3
Hematocrit (%)	28.20	31.40	45.00	39–49
MCV (fL)	86.00	85.60	78.50	80–100
MCH (pg)	26.00	27.50	25.50	26–34
MCHC (g/dl)	30.10	32.20	32.40	32–36
RDW (%)	16.30	15.60	15.20	11.6–14.6
Platelet count (10^3^/*μ*l)	351.00	261.00	241.00	150–440
C-reactive protein (mg/L)	5.33	232.70	—	0–3.0
Antinuclear antibodies	262.20	—	—	<20
Anti-Smith antibody (CU)	>693.50	—	—	<20
Anti-ribonuclear protein (CU)	>643.80	—	—	<20
Anti-SCL-70 (unit)	35.90	—	—	<20
Anti-dsDNA antibodies (IU/ml)	>666.90	>666.90	55.30	<27
Complement 3 (g/l)	0.13	0.40	0.79	0.9–1.8
Complement 4 (g/l)	0.01	0.06	0.16	0.1–0.4
Creatinine (mg/dl)	0.76	2.27	0.83	0.67–1.17
Creatinine kinase (U/l)	630.00	36.00	—	39–308
Protein/creatinine ratio (mg/g)	373.72	—	144.02	<200
Albumin/creatinine ration (mg/g)	—	1560.84	—	—
Protein (CSF) (mg/dl)	—	113.50	—	15–45
Glucose (CSF) (mg/dl)	—	45.40	—	40–70
Total leukocytes (CSF) (/MicroL)	—	4.00	—	—

## Data Availability

No data were used to support this study.
